# Perceived food intolerances can guide personalization of the FODMAP diet but not the choice of dietary intervention

**DOI:** 10.1002/jgh3.13017

**Published:** 2023-11-28

**Authors:** Dakota Rhys‐Jones, Chu K Yao, Peter R Gibson

**Affiliations:** ^1^ Department of Gastroenterology, Central Clinical School Monash University and Alfred Health Melbourne Victoria Australia

The evidence base for implementing a FODMAP diet to patients with irritable bowel syndrome (IBS) is now sufficiently robust that the diet has been incorporated in guidelines of countries across the world. However, the limitations of this approach include the need for a FODMAP‐trained dietitian to optimally deliver the diet and, in some regions of the world, the limited database of food content of FODMAPs and diverse dietary patterns.^1^ The appropriateness of implementing this diet in the Indian subcontinent was questioned in a study of self‐perceived food intolerances by Abraham *et al*., who suggested that it is more appropriate to ask the patient about their intolerances as a first‐step approach and use this to guide dietary advice to patients, rather than using a diet based on FODMAP composition alone.^2^ To apply such an approach, three key questions need to be addressed.

## Is this approach efficacious?

Unfortunately, no data were reported by Abraham *et al*. on outcomes from their suggested approach. Self‐initiated dietary restrictions based on perceived food intolerances do not have a good record of successful amelioration of chronic gastrointestinal symptoms. In a cross‐sectional study of patients with functional dyspepsia, gastrointestinal symptoms remained high despite 88% of patients following special diets, two‐thirds being low in FODMAPs.^3^ Subjects who identify gluten as the trigger for their symptoms, but do not have celiac disease—so‐called non‐celiac gluten/wheat intolerance—often follow a gluten‐free diet closely. However, the symptomatic response is often very poor.^4^ Further, multiple blinded rechallenge studies have shown that gluten is not the culprit in the vast majority, with fructans being the most likely culprit.^4^, ^5^

## What is the accuracy of perceived food intolerances?

The method used by Abraham *et al*. to identify food intolerances was quite reasonable—a “yes/no” answer from a list of primary ingredients (cereals, legumes, fruits, and vegetables), most of which are high in FODMAP content. Like in other populations, they found high rates of food intolerances. Thus, 45% of 400 healthy subjects and 72% of 204 IBS patients reported some sort of food intolerance, compared with rates between 70% and 80% in Western patient cohorts with IBS, functional dyspepsia. However, there were considerable discrepancies in the details. Wheat sensitivity was reported in the Indian cohort by no healthy subject and only 11% of the patients with IBS, and onions and garlic were a problem in less than 20%. This contrasts with the frequency of gluten or wheat avoidance that is reported to be as high as 10% globally^6^ and the observation that fructans found in foods containing wheat, onions, and garlic were major triggers of gut symptoms in blinded rechallenge studies of patients with IBS on a Western‐style diet.^7^ This discrepancy is unlikely due to gut effects of fructans not having pathogenic importance in Indian sufferers of IBS. Since the fructan effect is dose‐dependent,^7^ the dose of fructans consumed may be too low to be a problem in the Indian diet. This may be the case for wheat, particularly for the predominant rice eaters, but the dietary habits of the participants were not reported. However, onions and garlic are staple ingredients of many Indian dishes.^1^ The most likely explanation is that the association of fructan‐rich food components was just not recognized by many; for example, onions and garlic are seldom eaten in isolation.

Self‐identification of food intolerances is somewhat challenging when the offending food components are widespread in the diet. Patients can often be inaccurate when trying to untangle the dietary cause of symptoms, particularly when a large variety of high FODMAP ingredients are consumed together in several mixed dishes. This is true across the Asian continent, and particularly pertinent in India where multiple dishes are eaten as a meal with accompaniments such as chutneys and pickles.^1^ Indeed, such difficulties identifying food culprits provided the basis for the development of the FODMAP strategy in the first place.

Observations regarding intolerance to milk, which is an easily recognized component of the diet, are also instructive. Despite the high rates of hypolactasia in South Asian ethnicities, perceived intolerances to milk were similar between both healthy and IBS groups in the Indian cohort (30% healthy *vs* 23% IBS). The poor reliability of self‐reported intolerances to milk was highlighted in a somewhat confronting study in which a very high rate of dissimulation and an elevated score in the “lie scale” were found when the Minnesota Multiphasic Personality Inventory‐2 test was administered to subjects self‐describing as markedly milk intolerant.^8^ Many studies have reported overestimation of perceived *versus* true lactose intolerance. Hence, perceived food intolerances often overestimate the prevalence of a true reaction to food.

## Are there risks with this approach?

One concern about guiding dietary change according to perceived intolerances is that the diet may then be over‐restricted and, if not successful in ameliorating symptoms, additional restrictions are introduced, a phenomenon referred to as “diet stacking.” In patients with self‐perceived non‐celiac gluten/wheat sensitivity, it was somewhat disturbing that many often continue to be gluten‐free even when symptoms are poorly controlled or when gluten was shown in a blinded study not to induce their symptoms.^4^ The fear of gluten had been imbedded into their food belief system. In a Norwegian survey, 12% of people excluding or restricting food items that were self‐perceived as inducing symptoms were judged to have a nutritionally inadequate diet.^9^ Hence, designing dietary strategies based upon perceived food intolerances carries the risks of over‐restriction, nutritional inadequacy, and developing potentially irrational food‐belief systems.

## Strategies to assist implementing a FODMAP‐based diet in India

The motivation for Abraham *et al*.'s study was the difficulties in effectively and safely instituting a FODMAP dietary program in patients with IBS in India, where access to appropriately trained dietitians and extensive information on FODMAP food content are lacking. Although a randomized controlled study in India did report the efficacy of a low FODMAP diet, the main reasons for noncompliance were the nonfeasibility of preparing separate meals to their family and the limiting nature of the diet on staple foods such as onion, garlic, legumes, and lactose‐containing products.^10^ This highlights the difficulties in acceptability of the diet at a social, cultural, or religious level.

There are several ways of adapting a FODMAP dietary strategy to assist with its application the Indian subcontinent. First, simple measures in food preparation can lower oligosaccharide FODMAPs from some ingredients. For example, soaking legumes for extended periods or using tinned products if available (ensuring the liquid is discarded) can deplete the galacto‐oligosaccharides; the use of home‐prepared garlic/onion‐infused oils, or education on using garlic and onion in the beginning of the cooking process with oil/ghee and then removal can deplete the fructans. Second, the use of digestive enzymes to help improve tolerability of pulses could be implemented where these are accessible. Third, merging of the diet with advice given in the Ayurvedic dietary management of IBS and traditional dietary advice for IBS, such as reduction of foods such as garlic, onion, and pulses for bloating and abdominal pain (consequently reducing FODMAPs), a focus on regular meals, and avoiding fatty foods may improve acceptability. Fourth, a “FODMAP‐gentle” approach may be an attractive option for clinicians in India wishing to utilize FODMAPs with their patients with the current barriers in mind. This less researched approach describes only reducing a few commonly eaten foods that are particularly concentrated in FODMAPs and has been postulated as appropriate for groups for whom minimal dietary restriction is needed, such as children or the elderly. Fifth, the limited food composition data can be tackled by following the “best practical approach” restricting those foods that were known in FODMAP content.^10^ This might be considered a version of FODMAP‐gentle and resulted in sufficient symptomatic relief for patients in that clinical study. The success of a “moderate” approach of restricting foods with known FODMAPs has also been replicated in other countries where knowledge and use of the diet is not widespread. Finally, the foods commonly consumed in India continue to be updated on the Monash University FODMAP app, which can help clinicians to better guide their patients.

In conclusion, the simplicity of restricting the diet according to self‐perceived food intolerances in patients with IBS in India is attractive but lacks clinical outcome support to recommend it and carries potential risks of over‐restriction, nutritional inadequacy, and fear‐based food beliefs, all of which have been experienced and reported in Western cultures. Likewise, the optimal institution of a FODMAP diet with a culturally appropriate, personalized approach is challenging where available healthcare professionals trained in its delivery together with a food composition database are limited. In order to improve the delivery of efficacious diet therapies for patients with IBS, continued challenging of the paradigms currently followed in Western countries within India, such as that reported by Abraham *et al*., should be encouraged so that progress toward dietary solutions can be achieved.
